# Functional and genetic interactions of TOR in the budding yeast *Saccharomyces cerevisiae* with myosin type II-deficiency *(myo1Δ)*

**DOI:** 10.1186/1471-2121-13-13

**Published:** 2012-05-30

**Authors:** Glorivee Pagán-Mercado, Ednalise Santiago-Cartagena, Pearl Akamine, José R Rodríguez-Medina

**Affiliations:** 1 Department of Biochemistry, School of Medicine, Medical Sciences Campus, University of Puerto Rico, PO Box 365067, San Juan, PR, 00936-5067, USA

**Keywords:** *PKC1*, *SLT2/MPK1*, *WSC1*, *Tor2-21*, Fungal cell wall

## Abstract

**Background:**

Yeast has numerous mechanisms to survive stress. Deletion of myosin type II *(myo1Δ)* in *Saccharomyces cerevisiae* results in a cell that has defective cytokinesis. To survive this genetically induced stress, this budding yeast up regulates the *PKC1* cell wall integrity pathway (CWIP). More recently, our work indicated that TOR, another stress signaling pathway, was down regulated in *myo1Δ* strains. Since negative signaling by TOR is known to regulate *PKC1*, our objectives in this study were to understand the cross-talk between the TOR and *PKC1* signaling pathways and to determine if they share upstream regulators for mounting the stress response in *myo1Δ* strains.

**Results:**

Here we proved that TORC1 signaling was down regulated in the *myo1Δ* strain. While a *tor1*Δ mutant strain had increased viability relative to *myo1Δ*, a combined *myo1Δtor1*Δ mutant strain showed significantly reduced cell viability. Synthetic rescue of the *tor2-21*^*ts*^ lethal phenotype was observed in the *myo1Δ* strain in contrast to the *chs2*Δ strain*,* a chitin synthase II null mutant that also activates the *PKC1* CWIP and exhibits cytokinesis defects very similar to *myo1Δ,* where the rescue effect was not observed. We observed two pools of Slt2p, the final Mitogen Activated Protein Kinase (MAPK) of the *PKC1* CWIP; one pool that is up regulated by heat shock and one that is up regulated by the *myo1Δ* stress. The cell wall stress sensor *WSC1* that activates *PKC1* CWIP under other stress conditions was shown to act as a negative regulator of TORC1 in the *myo1Δ* mutant. Finally, the repression of TORC1 was inversely correlated with the activation of *PKC1* in the *myo1Δ* strain*.*

**Conclusions:**

Regulated expression of *TOR1* was important in the activation of the *PKC1* CWIP in a *myo1Δ* strain and hence its survival. We found evidence that the *PKC1* and TORC1 pathways share a common upstream regulator associated with the cell wall stress sensor *WSC1*. Surprisingly, essential TORC2 functions were not required in the *myo1Δ* strain. By understanding how yeast mounts a concerted stress response, one can further design pharmacological cocktails to undermine their ability to adapt and to survive.

## Background

The calcium-dependent protein kinase (Pkc1p) and target-of-rapamycin (TOR) signaling pathways are conserved in yeast and other fungi and are important for stress response and fungal survival. In addition to regulating growth and metabolic activity in normal cells, these pathways also regulate the cellular response to transient cell wall stress during the normal yeast life cycle, and during exposure to heat shock, cell wall damage, or other stressors that can compromise cellular integrity [1-3]. Our studies with myosin type II-deficient *(myo1Δ)* strains of the budding yeast *Saccharomyces cerevisiae,* which we have characterized previously as stress mutants, showed that the Pkc1p pathway is activated and essential for *myo1Δ* strain survival [4-6]. It has been our contention that this activation is due to cell wall stress caused by morphological abnormalities in the lateral cell wall and bud neck architecture [7,8]. In response to cell wall damage, heat shock, and other types of environmental stress, Rho1p activates the *PKC1* cell wall integrity pathway (CWIP), which in turn activates Slt2p (Mpk1p), the Serine/Threonine (Ser/Thr) MAPK at the end of this cascade [1-3]. This leads to transcriptional up regulation of cell wall-related genes by the Rlm1p transcription factor [9-12]. In addition to regulating the genetic program for cell wall integrity through the transcription factor Rlm1p [9,13,14], Slt2p may also modulate *PKC1* activity indirectly by a previously proposed feedback mechanism that phosphorylates and down regulates the Rho1p GDP-GTP Exchange Factor (GEF) Rom2p [15]. Rho1p also functions as the regulatory subunit of Fks1p, a β-1,3-glucan synthase for lateral cell wall fortification [16].

In prior studies, we have shown that similar to wild-type (wt) cells under stress conditions, the *myo1Δ* mutant (a genetically induced stress caused by the deletion of myosin II heavy chain that inhibits normal cytokinetic ring assembly) also activates the *PKC1* CWIP, but uses a different repertoire of genes [4,5]. Further characterization of the genes of the *myo1Δ* mutant at the post-transcriptional level showed that only a subset of cell wall integrity genes was activated. Thus, the *myo1Δ* mutant may serve as a simplified model for studying the cell wall stress response. Furthermore, we found that translation and ribosome biogenesis were down regulated in the *myo1Δ* strain [17]. This observation led us to investigate the role of TOR in the *myo1Δ* strain survival and how it may complement the reduced CWIP response.

Yeast TOR consists of two proteins - Tor1p and Tor2p - which are contained in two protein complexes TORC1 and TORC2 [18,19]. The TORC1 complex that is sensitive to rapamycin treatment contains proteins Tor1p or Tor2p, Kog1p, Tco89p and Lst8p [18,20-22]. TORC2 that is resistant to rapamycin treatment contains Tor2p, Avo1p, Avo2p, Avo3p, Bit61p, and Lst8p [18,20]. Recent subcellular localization studies showed that Tor1p was concentrated near to the vacuolar membrane while Tor2p was predominantly in punctuate structures near to the cytoplasmic surface of the plasma membrane [23]. Their differences in composition, sensitivity to rapamycin, and cellular localization support the idea that they function as two separate complexes [18,20,23]. TOR is important for nutrient sensing and is believed to play an important role in life span extension [24-27]. While TOR is conserved structurally and functionally from yeast to human, their roles are not biologically identical and warrant careful characterization of TOR from both species.

**Figure 1:**
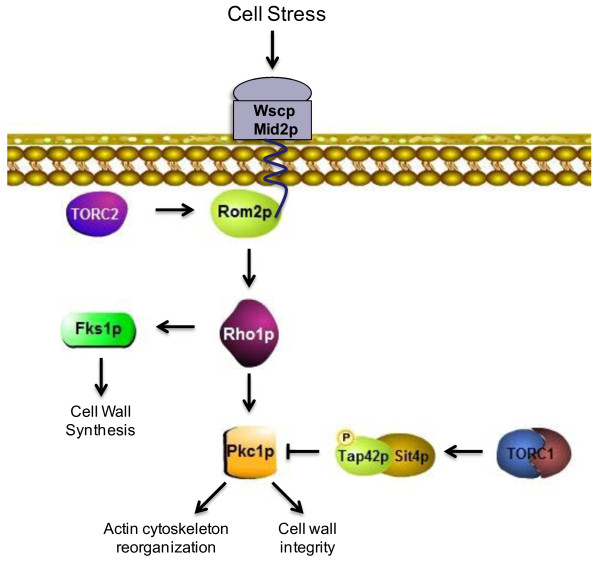
**General Description of the known interactions between the *PKC1-*Cell Wall Integrity and TOR Pathways in budding yeast.**

Rho1p is regulated by two mechanisms, a TOR-independent mechanism that is activated by cell wall stress (discussed above) and a separate TORC2-dependent mechanism that regulates actin cytoskeleton reorganization through the Rho1p-dependent activation of *PKC1 *[28]. In this latter pathway, Rom2p activity is indirectly modulated by the essential phosphatidylinositol kinase TORC2 via a GTPase switch consisting of Rho1p, Rho2p, Rom2p, and Sac7p (a GTPase activating protein of Rho1p) [29,30]. In short, the TORC2-dependent association of Rho1p (and Rho2p) with the Rom2p phosphatidylinositol-binding domain promotes Rom2p activation and downstream events [30,31]. In this manner Rom2p functions as the relay by which TORC2 regulates polarization of the actin cytoskeleton via Pkc1p. Therefore, functional interconnections between Pkc1p and TORC2 have been proposed through a mechanism integrated by the Rho1p-Rom2p complex [30,31] (Figure 1).

The stress sensor proteins Wsc1p, Wsc2p, Wsc3p, Mid2p and Mtl1p are involved in the activation of cell integrity signaling [2,32-37]. These cell surface sensors span the plasma membrane and are attached to the extracellular cell wall. The Mid2p homologue Mtl1p, that shares 50% sequence identity with Mid2p, appears to have a minor role in *PKC1* signaling [14]. These sensors react differently under specific stress conditions [37]. It has been reported that cells lacking *WSC1* are hypersensitive to drugs interfering with the cell wall and plasma membrane like Calcofluor white, Congo red, Caspofungin, Chlorpromazine and tea tree oil [1,38-40]. Additionally, Wsc1p responds to hypo-osmotic and alkaline pH conditions [39,41]. A *mid2*Δ mutant is hypersensitive to pheromone treatment, is hyperresistant to Calcofluor white, tea tree oil and Congo Red, and it senses acidic conditions and vanadate [32,37,39,40,42-44]. Wsc1p and Mid2p are also involved in the response to heat shock [2,33,35,45]. *WSC2* and *WSC3* act as suppressors of mutants defective in glycerol synthesis [37], while Mtl1p is associated with response to oxidative stress and glucose starvation [46,47]. The Wsc family of proteins and Mid2p have been shown to interact with specific signaling proteins that transmit stress signals from the fungal cell wall sensors to the Pkc1p and TOR signaling pathways. For example, Rom2p, the GEF that regulates Pkc1p, physically interacts with Wsc1p, Wsc2p, and Mid2p to activate the *PKC1* CWIP in the response to cell wall stress [36,48,49]. To define the nature of these signaling interactions in *myo1Δ* strains, we demonstrate here that TORC1 and Pkc1p activities were inversely correlated, which suggests cross-talk between the two pathways. Furthermore, we found that TORC1 was down regulated in *myo1Δ* strains by a mechanism that required expression of Wsc1p but not the other cell wall stress sensors. Surprisingly, Tor2p functions were not essential for survival in *myo1Δ* cells.

## Results

### TORC1 activity is down regulated in *myo1Δ* strains

**Figure 2:**
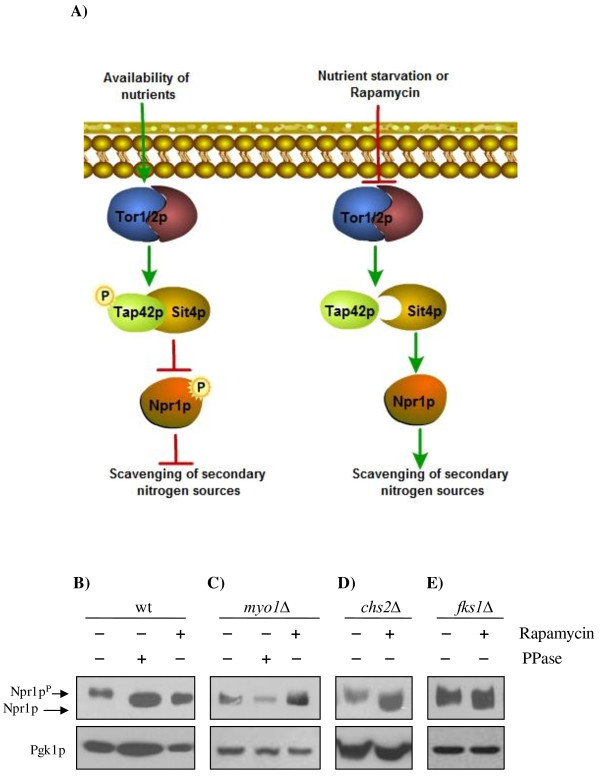
**The TORC1 pathway is down regulated in *myo1***Δ **strains but not in other cell wall stress models. A**) Schematic representation of the TOR signaling pathway and regulation of the phosphorylation state of Npr1p following inhibition of TOR by nutrient starvation or rapamycin. **B–E)** Western blot analysis of HA-*NPR1* showing the difference in electrophoretic mobility of phosphorylated Npr1p (Npr1p^P^) and the dephosphorylated form, Npr1p, following treatments with rapamycin, PPase or a genetic mutation of *MYO1* (see Methods for details). Arrows point to the expected positions of the Npr1p^p^-phosphorylated form (100 kiloDaltons, kDa) and Npr1p-dephosphorylated form (85 kDa). Protein extracts were analyzed from A) wild-type (wt) (10 μg), **B**) wild-type (wt) (10ug), **C**) * myo1Δ* (40ug), and **D**) *chs2Δ* (20ug),and **E**) *fks1Δ* (20ug).

To test the hypothesis that the TORC1 pathway was down regulated in the *myo1Δ* strain, we measured the levels of phosphorylated or dephosphorylated Npr1p to assess the TORC1 status [50]. TORC1 signaling regulates phosphorylation of the Ser/Thr protein kinase Npr1p at 22 potential phosphorylation sites [51,52]. At steady state, Npr1p is maintained inactive by phosphorylation [51] (Figure 2A, left diagram). Inhibition of TORC1 activity by nutrient starvation or application of the antiproliferative drug rapamycin results in dephosphorylation and subsequent activation of Npr1p by the protein phosphatase Sit4p [50,51,53] (Figure 2A, right diagram). To detect Npr1p in the *myo1Δ* strains, an expression plasmid containing a functional N-terminal hemagglutinin (HA)-tagged *NPR1* gene (HA-*NPR1*) was transformed into wt and mutant strains [54].

The phosphorylation state of Npr1p can be deduced from its relative electrophoretic mobility on SDS-polyacrylamide gel electrophoresis (SDS-PAGE) and detected by Western blot analysis using an anti-HA antibody. In wt whole cell extracts, Npr1p was detected as a slower-migrating band, which corresponds to the hyperphosphorylated form, Npr1p^P^ (Figure 2B, lane 1). Treatment of wt whole cell extracts with exogenous Calf Intestinal Alkaline Phosphatase (PPase) converted the slower-migrating band to a faster-migrating band which corresponds to the *in vitro* dephosphorylated form, Npr1p (Figure 2B, lane 2). Treatment of wt cell cultures with rapamycin produced a faster-migrating Npr1p band that co-migrated with the PPase treated band, consistent with the inhibition of TORC1 activity by rapamycin (Figure 2B, lane 3). This experiment established that the activity of TORC1 could be assessed indirectly by observing the relative electrophoretic mobility of Npr1p by SDS-PAGE [50,53].

**Figure 3:**
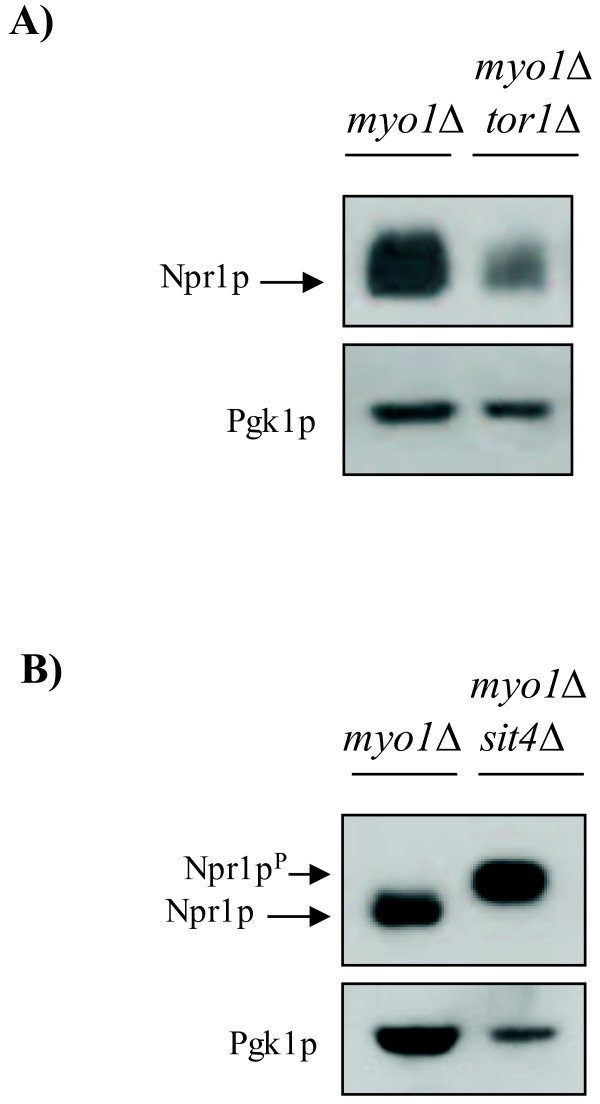
**TORC1 is down regulated and Npr1p dephosphorylation is Sit4p dependent in *myo1***Δ **strains.** Protein extracts from **A**) *myo1Δ* (40 μg), *myo1Δtor1*Δ (20 μg) and **B**) *myo1Δsit4*Δ (20 μg) mutant strains each expressing an HA-*NPR1* plasmid were analyzed by Western blot. Each membrane was probed with anti-HA and anti*-PGK1* antibodies. Pgk1p was used as a control. Arrows point to the expected positions of the Npr1p^p^-phosphorylated form and Npr1p-dephosphorylated form.

In *myo1Δ* whole cell extracts, no change in the electrophoretic mobility of Npr1p was observed as judged by the co-migration of Npr1p bands in extracts from rapamycin treated and untreated cells (Figure 2C, lanes 1 and 3). This result suggested that Npr1p is dephosphorylated at steady state in the *myo1Δ* strain. A *chs2*Δ strain (a chitin synthase II null mutant defective in contractile ring function) and an *fks1*Δ strain (a β-1,3-glucan synthase null mutant deficient in cell wall synthesis and maintenance) were incorporated as controls representing strains under cell wall stress [55,56]. In contrast, the electrophoretic mobility of Npr1p in *chs2*Δ (Figure 2D) and *fks1*Δ (Figure 2E) control strains behaved similar to the wt (Figure 2B). Phosphatase treatment did not change the electrophoretic mobility of Npr1p in the *myo1*Δ extracts supporting our hypothesis that TORC1 is down regulated (Figure 2C, lanes 1 and 2). Introduction of a *tor1*Δ mutation into the *myo1*Δ strain did not produce any change in the electrophoretic migration of Npr1p compared to the *myo1*Δ single mutant (Figure 3A). This was also consistent with the notion that TORC1 is inhibited in the *myo1*Δ strain. These observations suggest that the *myo1*Δ strain is distinct from the other cell wall stress models because despite the similarity between these strains which have the *PKC1* CWIP activated, the TORC1 activity was inhibited in the *myo1*Δ strain but not in the cell wall mutants tested (*chs2*Δ and *fks1*Δ) (see Discussion for details).

The *SIT4* gene encodes a protein phosphatase that is responsible for dephosphorylation of Npr1p *in vivo* during nutrient starvation (Figure 2A) [51]. TORC1 when active, phosphorylates Tap42p, which then binds and keeps Sit4p inactive [57]. Thus, Sit4p activity is negatively regulated by TORC1 [58]*.* In previous studies, Npr1p was shown to maintain the hyperphosphorylated state in a *sit4*Δ mutant treated with rapamycin indicating that its dephosphorylation was directly dependent on Sit4p activity [50,53]. To establish that dephosphorylation of Npr1p employs the same mechanism in *myo1*Δ, we conducted a Western blot analysis of Npr1p in a *myo1*Δ*sit4*Δ strain (Figure 3B). Absence of Sit4p activity in the *myo1*Δ strain resulted in the accumulation of the slow-migrating hyperphosphorylated Npr1p. This result supports that the *myo1Δ* dephosphorylation of Npr1p is via Sit4p.

**Figure 4:**
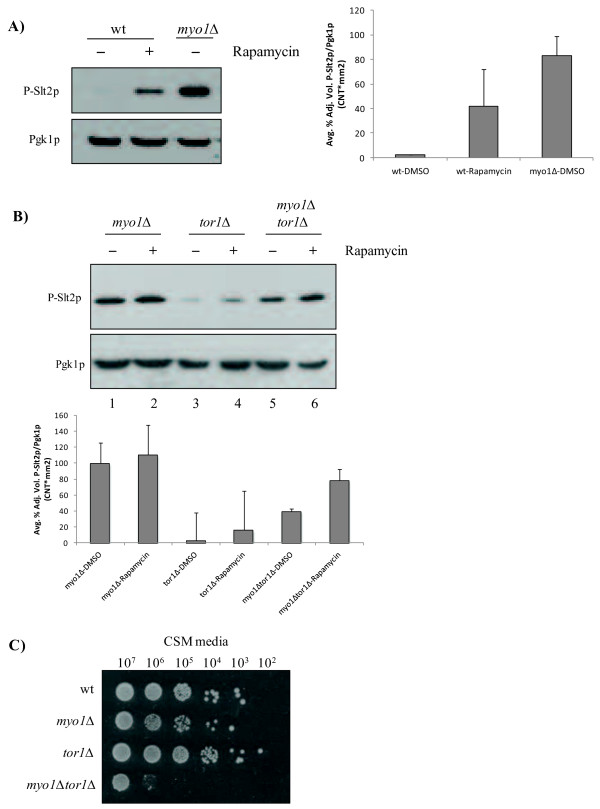
**Inverse correlation between TORC1 and *PKC1*activities. A**) The *PKC1* pathway was activated in wt cells upon inhibition of TORC1 with rapamycin. This pathway is constitutively activated in *myo1*Δ cells. **A**–**B**) All histograms show the ratio of the intensities of each P-Slt2p band relative to the intensity of its Pgk1p loading control, averaged from duplicate experiments. Error bars represent STDError mean. **B**) Steady state levels of hyper phosphorylated Slt2p (P-Slt2p, 55 kDa) were assayed by Western blot using equal amounts of protein extract (50 μg) from *myo1*Δ, *tor1Δ,* and *myo1Δtor1*Δ strains treated with rapamycin (+) or with DMSO alone (-). Pgk1p was used as a loading control. **C**) Limiting dilution growth assay on agar medium measuring relative viability of wt, *myo1Δ, tor1Δ,* and *myo1Δtor1*Δ strains. 10-fold dilutions are indicated at the top of the image (see Methods for details).

TOR signaling activity was previously reported to negatively regulate the *PKC1* CWIP [59] because rapamycin treatment resulted in up regulation of *PKC1* activity. Our new observations show that TORC1 was repressed in *myo1Δ* cells while we had previously shown that *PKC1* activity was up regulated in these strains [4,6]. To determine if the repression of TORC1 activity in *myo1Δ* cells is responsible for up regulation of the *PKC1* pathway, we treated *myo1Δ* cells with rapamycin and monitored Slt2p/Mpk1p hyperphosphorylation (referred to as P-Slt2p from here on) by Western blot analysis (see Methods). As previously reported, down regulation of TORC1 activity in wt cells treated with rapamycin resulted in up regulation of *PKC1*, reflected by an accumulation of P-Slt2p (Figure 4A). In untreated *myo1Δ* cells, there was an accumulation of P-Slt2p as described previously (Figure 4A). There was no significant increase in P-Slt2p levels following rapamycin treatment in these cells (Figure 4B, lanes 1 and 2). This observation supports the aforementioned result [59] that down regulation of TORC1 activity is correlated to the up regulation of *PKC1*.

To analyze this putative cross-talk further, we assayed the relative levels of P-Slt2p in a *myo1*Δ*tor1*Δ strain. In the absence of Tor1p, a *myo1*Δ*tor1*Δ strain maintained significant steady state levels of P-Slt2p at approximately 50% of the *myo1Δ*- levels (Figure 4B, lane 5), while treatment with rapamycin did not generate a significant change in these levels (Figure 4B, lane 6). A *tor1*Δ single mutant activated *PKC1* at low levels (Figure 4B, lanes 3 and 4).

To determine if these strains presented a growth defect we tested wt, *myo1Δ*, *tor1*Δ and *myo1Δtor1*Δ strains for cell viability using a serial dilution assay (Figure 4C). The *myo1Δ* strain exhibited a viability range similar to the wt strain. Surprisingly, the *tor1*Δ strain showed increased viability through the 10^2^ cells/ml range, consistent with previous studies that showed that Tor1p functions were not essential for cell viability. Despite having P-Slt2p present (Figure 4B, lane 5) the *myo1Δtor1*Δ strain presented a reduction in cell viability of approximately four orders of magnitude (Figure 4C, bottom row). Therefore, down regulation of TORC1 appears to be favorable to maintain viability in the *myo1Δ* strain while a complete absence of Tor1p in this strain is detrimental. These results imply that Tor1p may have a predominant role in the TORC1 functions with less activity attributed to the Tor2p in this complex. However, the residual activity in the TORC1 complex was essential for *myo1Δtor1*Δ strain survival because five days treatment with the IC50 of rapamycin (44nM) resulted in a 10-fold further reduction of growth (data not shown).

### Positive genetic interaction between *MYO1* and *TOR2*: lethality of a *tor2-21*^*ts*^ allele at 37°C is rescued by *myo1*Δ

**Figure 5:**
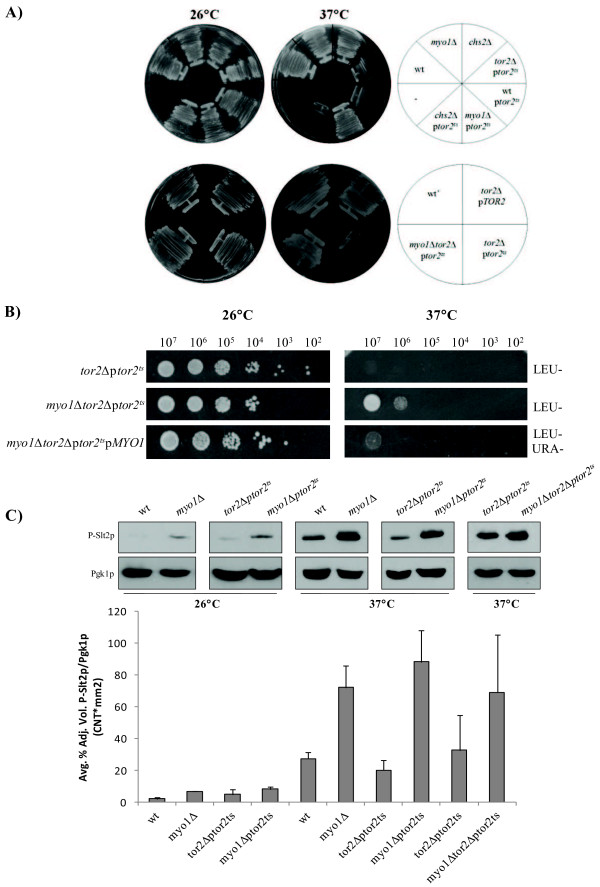
**Synthetic rescue of the *tor2-21***^***ts ***^**phenotype by*myo1***Δ**.** Assay for viability of yeast strains by growth at 26°C and 37°C. Strains tested were wt (YJR24), myo1Δ, *chs2*Δ, wt’ (JK9-3da), *tor2*Δ p*tor2*^*ts*^, wt p*tor2*^*ts*^, *myo1Δ* p*tor2*^*ts*^, *chs2*Δ p*tor2*^ts^, *tor2*Δ p*TOR2*, *myo1Δtor2*Δp*tor2*^*ts*^. **A**) Rescue of *tor2–21*^*ts*^ lethality at 37°C by *myo1Δ* in the YJR13 strain background (top) and SH121 strain background (bottom). **B**) Limiting dilution growth assay on agar medium measuring relative viability at 26°C and 37°C for *tor2*Δ p*tor2*^*ts*^, *myo1Δtor2*Δp*tor2*^*ts*^, and *myo1Δtor2*Δp*tor2*^*ts*^p*MYO1* strains. 10-fold dilutions are indicated at the top of the image (see Methods for details). **C**) Regulation of Slt2p phosphorylation in *myo1Δ* strains expressing the *tor2–21*^*ts*^ mutation at 37°C. Steady state levels of P-Slt2p in wt, *myo1*Δ, *tor2*Δ p*tor2*^*ts*^, and *myo1Δ* p*tor2*^*ts*^ were analyzed by Western blot as described previously from cultures grown at 26°C and 37°C. Pgk1p was used as a loading control. Histograms show the ratio of the relative intensities of each P-Slt2p band and its Pgk1p loading control, averaged from duplicate experiments. Error bars represent STD Error Mean.

TORC1 is not essential in wt [60] or *myo1Δ* strains. In contrast, TORC2 carries out essential functions in yeast cells that are not shared with TORC1 [21,22,61]. Growth at the permissive (26°C) and restrictive (37°C) temperatures was assayed for strains wt, *myo1Δ*, *chs2*Δ (each bearing a genomic copy of wild type *TOR2*), SH121 (a control *tor2*Δ strain that contains a plasmid-borne copy of a temperature^_^sensitive *tor2-21* allele, p*tor2*^*ts*^) and combination strains bearing also the p*tor2*^*ts*^ plasmid. Since TORC2 is responsible for essential cellular functions, the expected outcome was that repression of the *tor2-21*^*ts*^ allele at 37°C would be lethal in any strain. Parental strains and mutant strains bearing p*tor2*^*ts*^ were therefore assessed for growth on agar at 26°C and 37°C. As expected, the wt, *myo1Δ,* and *chs2*Δ strains did not exhibit temperature sensitive growth (Figure 5A, top row). The *tor2*Δ p*tor2*^*ts*^ (control), wt p*tor2*^*ts*^, and *chs2*Δ p*tor2*^*ts*^ strains were viable at 26°C and temperature sensitive for growth at 37°C indicating dominance of the *tor2-21*^*ts*^ mutation (Figure 5A, upper left and upper right respectively). In contrast, the *myo1Δ* p*tor2*^*ts*^ strain presented viable growth at both temperatures (Figure 5A, top row). The suppression of *tor2-21*^*ts*^ lethality by *myo1Δ* in this strain (YJR13) was also confirmed in the SH121 strain background (Figure 5A, bottom left and bottom right, respectively) to exclude genetic background effects. Growth was also assessed on Leucine dropout medium plates to ascertain that strains transformed with the p*tor2*^*ts*^ allele were expressing the plasmid, as observed by the normal growth at 26°C [Additional file 1, left plates], and again that the *tor2-21*^*ts*^ defect was suppressed in the absence of the *MYO1* gene at 37°C independently of the strain background [Additional file 1, right plates]. The positive genetic interaction between *myo1Δ* and *tor2-21*^*ts*^ mutations was reverted by complementation with a plasmid-borne copy of the wild type *MYO1* gene in *myo1*Δ*tor2*Δp*tor2*^*ts*^p*MYO1* (SH121) (Figure 5B) and also *myo1Δ*p*tor2*^*ts*^p*MYO1* (YJR13) strains (data not shown).

TORC2 has been shown to have a strong regulatory effect on *PKC1* activity in cell wall mutants [29,30]. To explain the observed synthetic rescue of *tor2-21*^*ts*^ lethality by *myo1Δ,* we conducted a Western blot analysis of P-Slt2p levels in the wt, *myo1Δ* (Figure 5C, box1), *tor2*Δ p*tor2*^*ts*^ and *myo1Δ* p*tor2*^*ts*^ strains (Figure 5C, box 2) at the permissive temperature. Wt and *tor2*Δ p*tor2*^*ts*^ strains showed similarly low P-Slt2p levels consistent with growth under non-stress conditions (Figure 5C, box 1 and box 2 respectively). The *myo1Δ* (Figure 5C, box1) and *myo1Δ* p*tor2*^*ts*^ (Figure 5C, box2) strains both presented a higher level of P-Slt2p relative to wt strains at 26°C, which was also consistent with previous observations that *PKC1* is activated in these strains (Figure 4A, lane 3).

To assess if rescue of *tor2-21*^*ts*^ lethality at the restrictive temperature by *myo1Δ* was accompanied by a change in *PKC1* activity levels, P-Slt2p was analyzed in whole cell extracts from cultures taken at 37°C (Figure 5C boxes 3 and 4). The temperature shift to 37°C produced an increase in P-Slt2p levels in all four strains attributable to the heat shock effect that is known to activate the *PKC1* pathway [1,62]. This suggests that there may exist two pools of Slt2p, one that is activated by the *myo1Δ* mutation and one that is activated (or phosphorylated) by the heat stress. Densitometric quantification and normalization of autoradiographs from duplicate experiments established that P-Slt2p levels in the* myo1Δ* p*tor2*^*ts*^ strain at 37°C were 5-fold higher than in the *tor2*Δ p*tor2*^*ts*^ strain yet were very similar to the *myo1Δ* single mutant strain at 37°C (Figure 5C, bottom panel*).* However, when we compared P-Slt2p levels between the *tor2*Δ p*tor2*^*ts*^ and *myo1Δtor2*Δp*tor2*^*ts*^ strain, there was no significant difference between them supporting that the rescue effect was not due to Tor2p-dependent P-Slt2p up regulation (Figure 5C, box 5) or differences in P-Slt2p levels.

### Evidence for a cross-talk between Pkc1p, TORC1, and cell wall stress sensor Wsc1p

**Figure 6:**
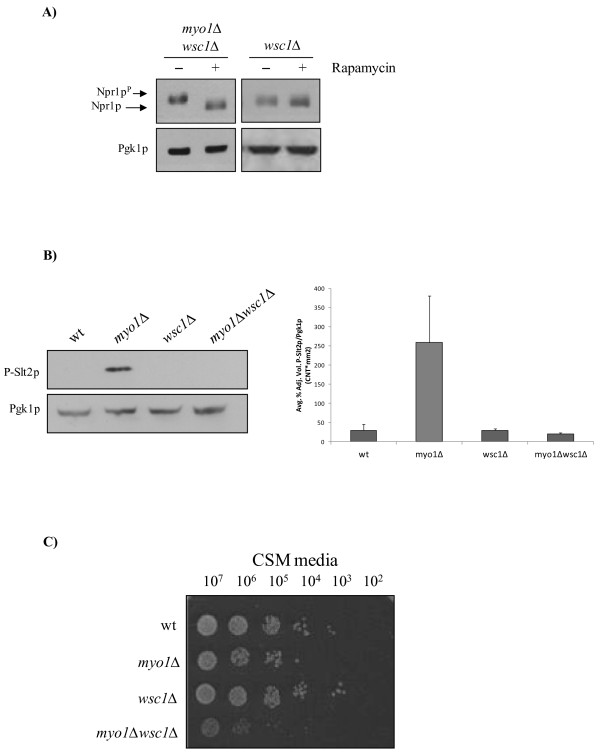
**Negative interaction between TORC1 activity and the Wsc1p cell wall stress sensor. A**) Western blot analysis of Npr1p electrophoretic mobility in *wsc1*Δ mutant strains (see methods for details). Whole cell protein extracts were prepared from *myo1Δ*(40 μg), *wsc1*Δ(20 μg) and *myo1Δwsc1*Δ(20 μg) strains expressing the *HA-NPR1* as described. Reactivation of TORC1 is observed in a *myo1Δwsc1*Δ strain and dephosphorylation occurs upon Inhibition of TORC1 by rapamycin. A *wsc1*Δ strain shows down regulation of TORC1. **B**) Western blot analysis of P-Slt2p levels in *wsc1*Δ mutant strains. 50 μg whole cell protein extracts were analyzed per lane. Histograms show the ratio of the relative intensities of each P-Slt2p band and its Pgk1p loading control, averaged from duplicate experiments. Error bars represent STDError mean. **C**) Limiting dilution growth assay on agar medium measuring relative viability of wt, *myo1Δ, wsc1*Δ and *myo1Δwsc1*Δ strains at 26°C. 10-fold dilutions are indicated at the top of the image (see Methods for details).

Inhibition of TOR functions activates multiple cell wall stress sensor proteins located in the plasma membrane that interact with signaling intermediates through their C-terminus in the cytoplasm [59]. Cell wall stress sensor proteins that belong to the Wsc family and also include the Mid2 proteins, signal positively to activate the *PKC1* CWIP [2,32-37]. Under cell wall stress conditions, Wsc1p, Wsc2p and Mid2p are reported to be the principal sensors responsible for activating this pathway [36,48,49]. Consistent with the notion that cell wall stress sensors may mediate the stress response in *myo1Δ* strains, we have presented evidence that the *PKC1* CWIP is activated and essential [4-6] and that TORC1 activity is down regulated in *myo1Δ* strains (this study). To test if cell wall stress sensor proteins could, by a cross-talk mechanism, be involved in down regulating the TOR pathway, the *myo1Δ* mutation was inserted in *wsc1*Δ, *wsc2*Δ, *wsc3*Δ and *mid2*Δ mutant strains by standard genetic techniques (see Methods). The single mutants (*wsc1*Δ, *wsc2*Δ, *wsc3*Δ, *mid2*Δ) and their corresponding double mutant strains (*myo1Δwsc1*Δ, *myo1*Δ*wsc2*Δ, *myo1*Δ*wsc3*Δ, *myo1*Δ*mid2*Δ) were tested for TORC1 activity and cell viability (only the results for Wsc1p are shown, Figure 6).

Relative TORC1 activity levels for the wt and *myo1Δ* strains were previously shown (Figures 2B and 2C respectively), while the results for *myo1Δwsc2*Δ, *myo1*Δ*wsc3*Δ and *myo1*Δ*mid2*Δ strains were also consistent with down regulated TORC1 activity in these strains (data not shown). In contrast, the *myo1*Δ*wsc1*Δ double mutant strain exhibited a result that was consistent with a fully active TORC1 (Figure 6A, lane 1) as judged by the relative decrease in electrophoretic mobility normally exhibited by Npr1p^p^, and the restored sensitivity of Npr1p^p^ electrophoretic mobility to rapamycin treatment (Figure 6A, lane 2)*.* Also, like the *myo1Δ* and *wsc1*Δ strains (Figure 6A, lanes 3 and 4)*,* preliminary results show that *wsc2*Δ, *wsc3*Δ, and *mid2*Δ single mutant strains (data not shown) exhibited a rapamycin-insensitive Npr1p electrophoretic mobility that was consistent with down regulation of TORC1. These results indicate that absence of these cell wall stress sensors represents a cell stress and supports the idea that they also play a role during normal cell growth. We therefore conclude from these results that Wsc1p may be associated with the regulation of TORC1 in both the wt and *myo1Δ* strains*.*

Because TORC1 and *PKC1* activities maintain an inverse relationship [59], we predicted that a re-activation of the TORC1 observed in the *myo1*Δ*wsc1*Δ strain would exert an inhibitory effect on the *PKC1* pathway. Consistent with this hypothesis, the *myo1*Δ*wsc1*Δ strain failed to activate the *PKC1* pathway as evidenced by undetectable levels of P-Slt2p relative to a *myo1Δ* single mutant where *PKC1* was activated (Figure 6B). Cell viability analysis revealed a reduction in growth of approximately one order of magnitude in the *myo1*Δ*wsc1*Δ double mutant strain relative to the *myo1*Δ strain and two orders of magnitude relative to wt and *wsc1*Δ strains that grew comparably well (Figure 6C).

**Figure 7:**
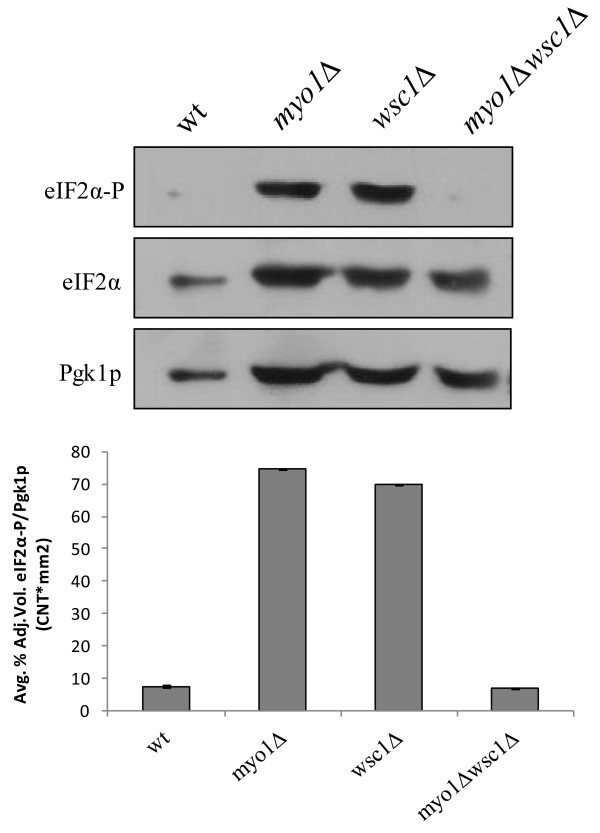
**Dephosphorylation of eIF2α-P confirms activation of TORC1 activity in *myo1Δwsc1***Δ**.** Western blot analysis of steady state levels of eIF2α and its phosphorylated form eIF2α-P was conducted with 50 μg per lane of whole cell protein extract derived from wt, myo1Δ, *wsc1*Δ and *myo1*Δ*wsc1*Δ strains. Pgk1p was used as a loading control. Histograms show the ratio of the relative intensities of each eIF2α band and its Pgk1p loading control, averaged from duplicate experiments. Error bars represent STD Error mean.

We then tested eIF2α phosphorylation levels as an additional readout of TORC1 status [63]. Previous studies showed that TORC1 down regulation in *myo1Δ* was evidenced by a large (2 fold) accumulation of phosphorylated eIF2α (eIF2α-P) produced by a TOR-dependent activation of Gcn2p protein kinase (Figure 7, top panel) [17]. The *myo1Δwsc1*Δ mutant combination that restored wild type electrophoretic mobility to Npr1p (Figure 6A) also restored eIF2α-P to its active unphosphorylated state, eIF2α (Figure 7, top panel, lane 4), thereby confirming that TORC1 was being reactivated in this mutant. Likewise, the accumulation of eIF2α-P in the *wsc1*Δ mutant confirmed that TORC1 activity was down regulated in this mutant (Figure 7, top panel, lane 3). Control extracts from the wt and *myo1Δ* strains were consistent with previously reported results [17]. Total eIF2α confirmed that the proposed changes in eIF2α-P were due to phosphorylation rather than a change in steady state levels of eIF2α (Figure 7, middle panel).

## Discussion

Yeast cells must respond rapidly and effectively to alterations in the environment in order to survive stressful conditions. These processes require the involvement of signal transduction pathways such as TOR and *PKC1*. The *PKC1* dependent CWIP is the first line of response to cell wall damage in the yeast *Saccharomyces cerevisiae*[13,14]. Transduction of the signal begins with the cell wall stress sensor proteins Wsc1p and Mid2p at the plasma membrane and proceeds through Rom2p and Rho1p to the *PKC1* CWIP that ends with activation of the MAP kinase, Slt2p [13,48] (Figure 1). Downstream, the transcription factor Rlm1p activates nuclear genes involved in cell wall synthesis and remodeling to produce a cell wall stress response that increases the survival potential of the yeast cell [9,10]. We have shown in prior studies that the *PKC1* pathway is continuously activated in *myo1Δ* strains [4,6]. This response is further characterized here in the *myo1Δ* strains. In addition to the up regulation of the CWIP we found that TORC1 was down regulated to enhance cell survival and we provide evidence of cross-talk between the two signaling pathways.

Npr1p is a protein kinase that regulates the amino acid permease Gap1p to transport secondary nitrogen sources into the cell for the restoration of amino acid precursor levels and protein synthesis [51,64,65]. When TORC1 is down regulated by nutrient starvation or rapamycin treatment, Npr1p becomes dephosphorylated by the protein phosphatase Sit4p, thereby activating its biochemical function [50,51,53] Furthermore, inactivation of TORC1 results in downregulation of ribosome and protein synthesis [59]. When we assayed the relative status of TORC1 activity in a *myo1Δ* strain, we observed that Npr1p was maintained in the dephosphorylated state and demonstrated that the Npr1p phosphorylation state was directly dependent on TORC1 and Sit4p activities. Therefore, we established that the TORC1 complex is found in a predominantly inactive state in the *myo1Δ* strain. The implications of such a metabolic state led us to believe that the survival of this strain is directly linked to this observation. However, the complete absence of Tor1p by genetic deletion (*tor1*Δ) was detrimental for survival of the *myo1Δ*strain, supporting that a precise level of TORC1 activity must be maintained for its survival. Furthermore, complete inhibition of TORC1 activity by rapamycin treatment of a *myo1Δtor1*Δ strain was lethal for growth, further supporting the idea that minimal levels of TORC1 activity are essential. Conversely, the *tor1*Δ single mutant was shown to acquire increased fitness, which was consistent with the proposed role of mTOR and TOR in regulating longevity and replicative life span extension respectively [24-27].

**Figure 8:**
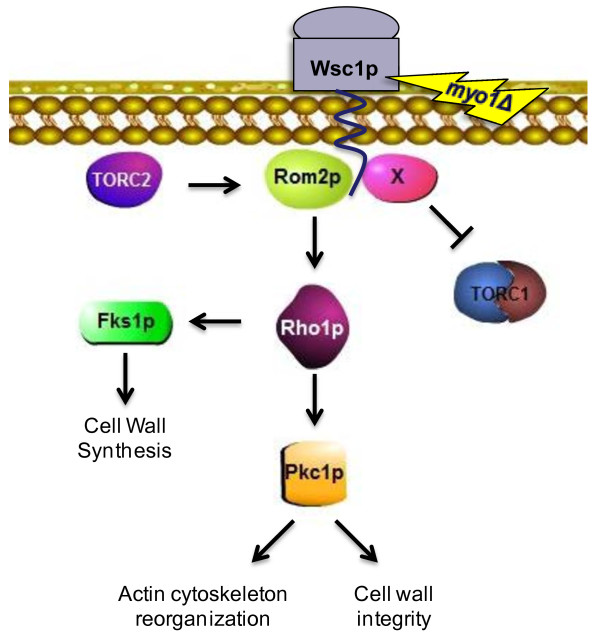
**Schematic representation of the proposed regulation of TOR and Pkc1p in the *myo1***Δ **strain.** The *myo1*Δ deficiency (yellow ray) creates a cell wall stress signal that is transduced through the Wsc1p stress sensor from the Rho1p-GEF Rom2p to Rho1p which activates the *PKC1* CWIP. Activation of Rho1p also leads to activation of Fks1p activity and cell wall synthesis. Regulation of cell wall integrity and actin cytoskeletal reorganization by Pkc1p could explain the synthetic rescue effect of *myo1*Δ in a *tor2–21*^*ts*^ strain. A novel negative regulation of TORC1 by Wsc1p was observed in the *myo1*Δ strain. We propose that the negative regulation of TORC1 by Wsc1p involves a novel interacting protein of Wsc1p labelled here as “X”.

It was previously known that the TORC1 pathway plays a role in the response to cell wall stress by a negative regulation of the *PKC1* CWIP under nutrient rich conditions [59] (Figure 1). Under *myo1Δ* conditions, we observed a clear inverse biochemical correlation between TORC1 and Pkc1p activities. This raised the question, is there a common upstream regulator of the two pathways? Our results are strongly suggestive that Wsc1p acts as a common upstream regulator of TORC1 and *PKC1* by exerting a positive role in the activation of the *PKC1* CWIP and a negative role in the down regulation of TORC1 by an unknown mechanism. We do not propose that TORC1 and Wsc1p interact directly. Most likely, the mechanism involves an undetermined protein interactor of Wsc1p (labeled “X” in Figure 8). Recently, we have identified several novel Wsc1p interacting proteins (unpublished results) and suspect that some may function as a signaling intermediate between Wsc1p and TORC1. However, pending future studies, it is not known whether or not these Wsc1p interactors inhibit TORC1.

Our findings showed that *chs2*Δ and *fks1*Δ mutant strains strongly activated the *PKC1* CWIP yet maintained normal TORC1 activity levels. We are therefore confronted with variable signaling outputs exiting from the cell wall stress sensors. In particular, disruption of cell wall integrity by these mutants leads to activation of the *PKC1* CWIP; however, only the disruption of cytokinesis in a *myo1Δ* strain leads to both activation of the *PKC1* CWIP and down regulation of TORC1 (Figure 8). This finding is consistent with the transcriptional profiles we have determined previously, where *myo1Δ* only regulated half of the CWIP fingerprint genes, while *fks1*Δ and *chs2*Δ profiles were more like other cell wall damage profiles [4,5]. This reinforces the idea that *myo1*Δ activates a cell signaling program that is distinct from other cell wall mutants. We propose that in addition to its filament assembly function, the tail domain may serve as a scaffold (or guide) for the assembly of interacting protein complexes at the cytokinetic ring that are important for myosin function [66]. Therefore, disruption of these putative protein assemblies by a genetic deletion of the *MYO1* gene may activate the cell wall stress sensors Wsc1p and Mid2p in a different manner than in the *chs2*Δ and *fks1*Δ mutants. The non-muscle myosin heavy chain (Myo1p) of budding yeast has been shown to have independent functions associated with the head and tail domains of the protein [67]. The tail domain contains a Minimum Localization Domain (MLD) that is sufficient to target the myosin heavy chain to the bud neck independently of the actin-binding site that is encoded within the head domain [68]. Therefore, despite the common activation of the *PKC1* CWIP among the *myo1*Δ*, chs2*Δ and *fks1*Δ mutant strains, we hypothesize that the inhibition of TORC1 by Wsc1p is unique to the *myo1*Δ mutant and may be triggered by the disruption of specific protein-protein interactions in the putative Myo1p scaffold at the cytokinetic ring.

The final question that arises from these results is, how does the *myo1*Δ mutant rescue *tor2-21*^*ts*^ lethality? Strains that carry the temperature-sensitive gene of *TOR2* (*tor2*^*ts*^) arrest growth at the restrictive temperature (37°C). This lethality is thought to be caused by the lack of TORC2 activity, decreased *RHO1* activation [21], the lack of actin organization and cell lysis probably due to cell wall defects [69]. The lethality has been shown to be rescued in several different ways. One way is by growth on nonfermentative carbon sources (i.e. raffinose) but not by nonfermentable carbon sources (i.e. glycerol or ethanol) [69]. A second way in which *tor2*^*ts*^ lethality can be circumvented is by treatment with agents that cause cell integrity stress (i.e. 0.005% SDS) [30]. A third way in which the *tor2*^*ts*^ lethality can be rescued is by the osmotic stabilizer, sorbitol, again suggesting that the cell wall is somehow compromised. Finally, there are several genes that have been shown to suppress the lethality of *tor2*^*ts*^ lethality. One example is yeast *PAS* kinase overexpression (a gene involved in glucose partitioning in the cell) which is thought to suppress the *tor2*^*ts*^ lethality by *RHO1*-dependent activation of *PKC1* and actin rearrangement, activation of *FKS1* and cell wall synthesis, or both [69]. These observations have lead to the idea that cell growth and survival is a product of signals derived from cell integrity and nutrient availability [69]. In this work we provide evidence for rescue of *tor2*^*ts*^ lethality by the deletion of the *MYO1* gene. We propose that *myo1*Δ rescues the *tor2*^*ts*^ lethality by invoking both strategies described above, namely, by activating a starvation type response (TOR) and the cell wall integrity pathway (*PKC1* CWIP), most likely through the reorganization of the actin cytoskeleton. However, unlike the results of Cardon et al. [69] where the essential Rho1p GEF was Rom2p, the roles of Rom1p and Rom2p appear to be redundant for the proposed *myo1*Δ rescue mechanism (data not shown).

## Conclusions

We have shown that cross-talk between the *PKC1* and TOR signaling cascades occur under the *myo1Δ* stress condition. TORC1 activity was found to be inversely correlated with activation of the *PKC1* pathway while both Tor1p and Pkc1p act as positive regulators of viability in the *myo1*Δ strain. Synthetic rescue of *tor2-21*^*ts*^ lethality by *myo1Δ* points to the *PKC1-*dependent reorganization of the actin cytoskeleton as the possible rescue mechanism. The data presented supports that in addition to its known role in signaling to the *PKC1* CWIP, Wsc1p may also function as an upstream regulator of TORC1.

## Methods

### Strains and media

*Saccharomyces cerevisiae* strains used in this study are listed in Table 1. Dr. Brian C. Rymond kindly provided the *TOR1*, *SIT4* and *WSC1* null mutation strains. The *myo1Δtor1*Δ, *myo1Δsit4*Δ, *myo1Δwsc1*Δ and *myo1Δtor2*Δp*tor2*^*ts*^ double mutants were constructed by a disruption of the *MYO1* gene with a *HIS5* module by homologous recombination using a PCR based method. Strains *tor2*Δp*TOR2* and *tor2*Δp*tor2*^*ts*^ were kindly provided by Dr. Michael N. Hall. The composition of complete synthetic media (CSM) for wild type cells was complemented with 2% Glucose and 1X Nitrogen base without amino acids. The composition of rich medium (YPD)/G for *tor1Δ, sit4Δ,* and *wsc1*Δ strains was complemented with 2% Glucose and 200ug/mL G418 (Geneticin). Histidine dropout media (CSM HIS-) was used for double mutant lacking the *MYO1* gene; while Leucine dropout media (CSM LEU-) was used for the maintenance of plasmids, each one complemented with 2% Glucose and the appropriate nitrogen base without amino acids. Cultures were grown overnight at 26°C to mid-logarithmic phase with an optical density between 0.5–0.8 AU (OD_600_) with continuous shaking at 225 rpm. A 1 mg/mL stock solution of rapamycin (SIGMA) was dissolved in the drug vehicle 100% DMSO. To inhibit TORC1, cultures were treated with rapamycin for 1 h with drug vehicle alone (DMSO) 100% or with a half maximal (50%) growth inhibitory concentration of rapamycin at a final concentration of 60nM (54.85 ng/mL) for wt cells and 44nM (36.56 ng/mL) for mutant cells, prior to harvesting. Cultures bearing plasmid p*tor2*^*ts*^ were grown to mid-log phase in CSM LEU- medium, and were then diluted to an OD_600_ of 0.5 in pre-warmed media. Cultures were shaken at 225 rpm at 37°C for 1 h.

**Table 1:** Strains used in this study

**Strain**	**Genotype**	**Source**
YJR12,YJR24_1 (wild type, wt)	*MAT* α trp1 ura3 leu2-3 his3Δ1 met- ADE + ARG + cyh^R^	Lab. Strain
JK9-3da (wt’)	*MAT* a leu2-3,112 trp ura3 rmel his4 HMLa	M. Hall
YJR13 (*myo1*Δ)	*MAT* a trp1 ura3 leu2-3 his3Δ1 met- ADE + ARG + cyh^R^*myo1Δ*::*HIS5+*	Lab. Strain
YFR22 (*fks1*Δ)	*MAT* α trp1-289 ura3-52 leu2-3, 112 his3Δ1 ADE + ARG + cyh^R^*fks1*Δ::*kanMX4*	F. Rivera
YFR23 (*chs2*Δ)	*MAT* α trp1-289 ura3-52 leu2-3, 112 his3Δ1 ADE + ARG + cyh^R^*chs2*Δ::*kanMX4*	F. Rivera
YJR066W (*tor1*Δ)	*MAT* α his3Δ1 leu2Δ0 lysΔ0 ura3Δ0 *tor1Δ::kanMX4*	B. Rymond
YGP1 (*myo1*Δ*tor1*Δ)	*MAT* α his3Δ1 leu2Δ0 lysΔ0 ura3Δ0 *tor1Δ::kanMX4, myo1Δ::HIS5+*	This study
YOR008C (*wsc1*Δ)	*MAT* α his3Δ1 leu2Δ0 lysΔ0 ura3Δ0 *wsc1Δ::kanMX4*	B. Rymond
YES1 (*myo1*Δ*wsc1*Δ)	*MAT* α his3Δ1 leu2Δ0 lysΔ0 ura3Δ0 *wsc1Δ::kanMX4, myo1Δ::HIS5+*	E. Santiago
MH346-1a/pJK3-3	JK9-3da ade2 tor2::ADE2/pSEY18::*TOR2*	M. Hall
SH121 (*tor2*Δ p*tor2*^ts^)	JK9-3da ade2 tor2::ADE2/YCplac111::*tor2-21*^*ts*^	M. Hall
YGP5 (*myo1*Δ*tor2*Δp*tor2*^ts^)	JK9-3da ade2 tor2::ADE2/YCplac111::*tor2-21*^*ts*^*myo1*Δ::*HIS5+*	This study
YJR12/pEJ23 (wt pHA-*NPR1*)	*MAT* α trp1 ura3 leu2-3 his3Δ1 met- ADE + ARG + cyh^R^, pHA-*NPR1*	This study
YJR13pEJ23 (*myo1Δ* pHA-*NPR1*)	*MAT* a trp1 ura3 leu2-3 his3Δ1 met- ADE + ARG + cyh^R^, *myo1*Δ::*HIS5* + pHA-*NPR1*	This study
(YFR22pEJ23) (*fks1*Δ pHA-*NPR1*)	*MAT* α trp1-289 ura3-52 leu2-3, 112 his3Δ1 ADE + ARG + cyh^R^*fks1*Δ::*kanMX4*, pHA-*NPR1*	This study
YFR23pEJ23 (*chs2*ΔpHA-*NPR1*)	*MAT* α trp1-289 ura3-52 leu2-3, 112 his3Δ1 ADE + ARG + cyh^R^*chs2*Δ::*kanMX4*, pHA-*NPR1*	This study
YJR066WpEJ23 (*tor1*ΔpHA-*NPR1*)	*MAT* α his3Δ1 leu2Δ0 lysΔ0 ura3Δ0 *tor1Δ::kanMX4*, pHA-*NPR1*	This study
YGP1pEJ23 (*myo1*Δ*tor1*Δ pHA-*NPR1*)	*MAT* α his3Δ1 leu2Δ0 lysΔ0 ura3Δ0 *tor1Δ::kanMX4 myo1*Δ*::HIS5+*, pHA*-NPR1*	This study
YDL047W pEJ23 (*sit4*ΔpHA-*NPR1*)	*MAT* α his3Δ1 leu2Δ0 lysΔ0 ura3Δ0 *sit4Δ::kanMX4*, pHA-*NPR1*	This study
YGP3pEJ23 (*myo1Δsit4*Δ pHA-*NPR1*)	*MAT* α his3Δ1 leu2Δ0 lysΔ0 ura3Δ0 *sit4Δ::kanMX4 myo1*Δ::*HIS5*+, pHA-*NPR1*	This study
YOR008C pEJ23 (*wsc1*ΔpHA-*NPR1*)	*MAT* α his3Δ1 leu2Δ0 lysΔ0 ura3Δ0 *wsc1Δ::kanMX4,* pHA*-NPR1*	This study
*YES1*pEJ23 (*myo1*Δ*wsc1*Δ pHA-*NPR1*)	*MAT* α his3Δ1 leu2Δ0 lysΔ0 ura3Δ0 *wsc1Δ::kanMX4 myo1*Δ::*HIS5+*, pHA-*NPR1*	This study
YJR24_1/YCplac111 (wt p*tor2*^*ts*^)	*MAT* α trp1 ura3 leu2-3 his3Δ1 met- ADE + ARG + cyh^R^, YCplac111::*tor2-21*^*ts*^	This study
*YJR13/*YCplac111 (*myo1*Δ p*tor2*^ts^)	*MAT* a trp1 ura3 leu2-3 his3Δ1 met- ADE + ARG + cyh^R^*myo1*Δ::HIS5+, YCplac111::*tor2-21*^*ts*^	This study
*YFR23/p*YCplac111 (*chs2*Δp*tor2*^ts^)	*MAT* α trp1-289 ura3-52 leu2-3, 112 his3Δ1 ADE + ARG + cyh^R^*chs2*Δ::*kanMX4*, YCplac111::*tor2-21*^*ts*^	This study
YJR13YCplac111pRS316*MYO1* (*myo1*Δp*tor2*^ts^p*MYO1*)	*MAT* a trp1 ura3 leu2-3 his3Δ1 met- ADE + ARG + cyh^R^*myo1*Δ::HIS5+, YCplac111::*tor2-21*^*ts*^ pRS316*MYO1*	This study
YGP5pRS316*MYO1* (*myo1*Δ*tor2*Δp*tor2*^ts^p*MYO1*)	JK9-3da ade2 tor2::ADE2, *myo1*Δ::*HIS5+,* YCplac111::*tor2-21*^*ts*^ pRS316*MYO1*	This study

### Plasmid and genetic techniques

Plasmid pHA-*NPR1* (pEJ23) consists of YEplac181 (*LEU2)* expressing a functional N-terminally HA-tagged *NPR1* under its own promoter [53], kindly provided by Dr. Estela Jacinto. Plasmid YCplac111::*tor2-21*^*ts*^ (*LEU2*) containing a temperature sensitive *tor2-21*^*ts*^ allele [21] was kindly provided by Dr. Michael N. Hall. *Escherichia coli* strain DH5α was used for the propagation and isolation of plasmids. Yeast transformations were performed by the Lithium acetate procedure. Yeast plasmid DNA was isolated by an adaptation from the QIAGEN QIAprep Spin miniprep kit.

### Western blot analysis

Whole yeast cell protein extracts were prepared by harvesting and lysing cell cultures by vortexing with glass beads for 20 s with 3 min intervals on ice (repeated 3 times). Lysis buffer contained 50 mM Tris–HCl pH 7.5, 10% Glycerol, 1% TritonX-100, 0.1%SDS, 150mN NaCl, and 5 mM EDTA, supplemented with 5X Protease Inhibitor Cocktail (50X stock; Roche) and 10 mM PMSF. Cell lysates were centrifuged at 13,000 rpm for 10 min at 4°C; the supernatant was removed and quantified using the DC Protein Assay method (Bio-Rad, Hercules, CA).

Whole protein extracts were denatured at 95°C for 5 min, separated on 10% SDS-polyacrylamide gels and transferred to nitrocellulose membrane at 0.37 Amps for 1 h at 4°C in a Mini Trans Blot Cell (Bio-Rad, Hercules, CA). Npr1p was consistently expressed more abundantly in wt cells than in any of the mutant cells. Therefore, the loading volumes were adjusted accordingly. The reason for these differences in Npr1p levels between strains is not known, but it has been speculated that it could be due to differences in the stability of the protein [53]. For analysis of HA-*NPR1,* membranes were probed with anti-HA rat monoclonal antibody (3 F10, Roche, 1:1000) in blocking solution containing 0.5% Western Blocking Reagent (Roche) diluted in 1X TBS (Tris Buffered Saline, Sigma Aldrich) at 4°C overnight and washed in 1X TBS/0.1% Tween-20 (TBS/T) (Sigma Aldrich). Membranes were counter-probed with a Horseradish Peroxidase (HRP) conjugated secondary Goat anti-rat IgG antibody (Pierce, 1:5000). For phosphorylated Slt2p (P-Slt2p), membranes were incubated with anti-phospho-p44/42 MAPK rabbit monoclonal antibody (Cell Signaling, 1:1000) in 5% BSA (Bovine Serum Albumin, Sigma Aldrich) plus TBS/T buffer at 4°C overnight. HRP-conjugated secondary antibody was Goat anti-rabbit IgG antibody (Pierce, 1:10000) diluted in blocking solution. For analysis of phosphorylated eukaryotic Initiation Factor α (eIF2α-P), the membrane was incubated with anti-phospho-eIF2α polyclonal antibody (Invitrogen, 1:1000) in blocking solution at 4°C overnight. Membranes were stripped and reprobed with a rabbit polyclonal antibody that recognizes both the phosphorylated and unphosphorylated forms of eIF2α (eIF2α)(kindly provided by Dr. Thomas E. Dever). HRP-conjugated secondary antibody was Goat anti-rabbit IgG antibody (Pierce, 1:10000) diluted in blocking solution. Membranes were also probed with a mouse monoclonal antibody against Phosphoglycerate kinase (Pgk1p) (Molecular Probes, Invitrogen, 1:500) as a loading control.

Proteins were detected using a chemiluminescent substrate (SuperSignal West Pico, Thermo Scientific), and membranes were exposed to X-ray film, which were then scanned with a Molecular Imager FX Pro Plus (Bio-Rad, Hercules, CA). Digital image intensity was quantified using Quantity One 4.5.2 software (BioRad). Protein bands were quantified according to the ratio of the intensity of the test protein relative to the intensity of its Pgk1p loading control. The obtained values were averaged from duplicate experiments. Quantitative units were expressed as CNT*mm^2^ or Contour Quantity. This is described as the sum of the intensities of all the pixels within the band boundary multiplied by the area of each pixel (Quantity One, Bio-Rad). Error bars represent the Standard Error of the mean (STDError mean), calculated as the standard deviation (STDEV)/Square root (SQRT) of the count.

### Alkaline phosphatase (PPase) treatment of protein extracts

To generate dephosphorylated proteins, 50 μg of whole yeast cell protein extract were incubated with 50U (1U/μg) of Calf Intestinal Alkaline Phosphatase (CIP or PPase, New England Biolabs) in the presence of 1X CIP buffer (10X NEB 3, New England Biolabs) and 5X Protease Inhibitors cocktail, EDTA Free (50X stock, Roche) for 30 min at 37°C. Samples were denatured at 95°C for 5 min and subjected to SDS-PAGE and Western blot analysis.

### Viability assay

Wt, *myo1Δ, tor1*Δ, *myo1Δtor1*Δ, *wsc1*Δ and *myo1*Δ*wsc1*Δ strains were grown to OD_600_ between 0.5–0.8 AU at 26°C with continuous shaking at 226 rpm. 5uL of serial dilutions ranging from 1x10^7^–1x10^2^ cells/mL were spotted onto CSM or selection media agar plates containing 2% Glucose and 1X Nitrogen base. Plates were incubated at 26°C to observe growth after three days of incubation. Strains expressing the temperature sensitive *tor2-21*^*ts*^ mutation were streaked on CSM or selection media agar plates, and were incubated at 26°C and 37°C for 2.5 days.

## Competing interests

The authors declare that they have no competing interests.

## Authors’ contributions

GP-M performed genetic knockout experiments, Western blotting, growth assays, data analysis and interpretation, and writing sections of the manuscript. ES-C participated in genetic knockout and Western blot experiments of WSC strains and growth assays. PA contributed to the data analysis and interpretation and to the writing and revision of sections of the manuscript. JRR-M as principal investigator, conceived the study, designed experiments, carried out data analysis and interpretation, wrote and revised the manuscript. All authors read and approved the final manuscript.

## Supplementary Material

Additional file 1 **Pagán-Mercado, Santiago-Cartagena, Akamine, and Rodríguez-Medina.** Assay for viability of yeast strains by growth at 26°C and 37°C in Leucine-deficient dropout agar medium. Strains wt (YJR24), *myo1Δ*, *chs2*Δ, wt’ (JK9-3da), *tor2*Δ p*tor2*^*ts*^, wt p*tor2*^*ts*^, *myo1Δ* p*tor*^*ts*^, *chs2*Δ p*tor2*^ts^, *tor2*Δ p*TOR2*, *myo1Δtor2*Δp*tor2*^*ts*^ were tested for presence of the p*tor2*^*ts*^ plasmid containing the *LEU2* marker. Click here for file
