# Supplementary figures and images for: Functional and genetic interactions of TOR in the budding yeast Saccharomyces cerevisiae with myosin type II-deficiency (myo1Δ)
Source: BMC Cell Biol. 2012 May 30;13:13. doi: 10.1186/1471-2121-13-13 (PMC3470973; doi:10.1186/1471-2121-13-13)

Additional file:

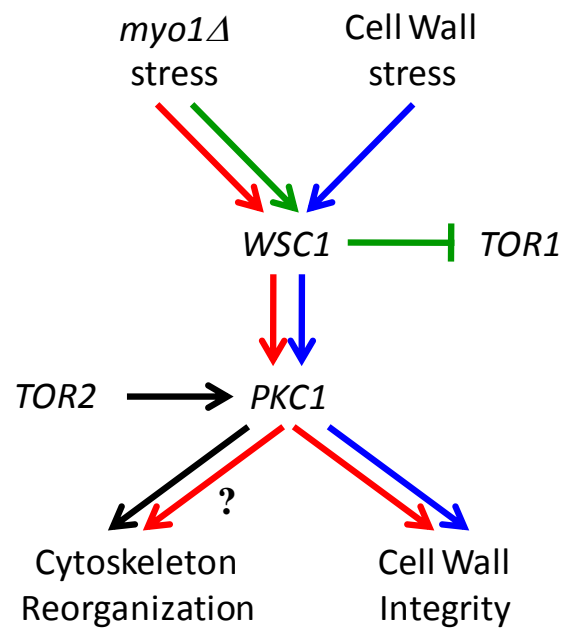

Supplement: Additional file 1 — Pagán-Mercado, Santiago-Cartagena, Akamine, and Rodríguez-Medina. Assay for viability of yeast strains by growth at 26°C and 37°C in Leucine-deficient dropout agar medium. Strains wt (YJR24), myo1Δ, chs2Δ, wt’ (JK9-3da), tor2Δ ptor2ts, wt ptor2ts, myo1Δ ptorts, chs2Δ ptor2ts, tor2Δ pTOR2, myo1Δtor2Δptor2ts were tested for presence of the ptor2ts plasmid containing the LEU2 marker. [file 1471-2121-13-13-S1.pdf]
